# New York Medical and Surgical Society

**Published:** 1854-03

**Authors:** Charles M. Allin

**Affiliations:** Secretary


New York Medical and Surgical Society.—Extracts from the Minutes.—By Charles M. Allin, M. D., Secretary.—Dr. Van Buren related a case of congenital syphilis, presenting unusual tertiary symptoms. The patient was a child, fourteen months old, not yet weaned, and well nourished. There was a large periosteal tumor in the right temporal region, and another upon the left ulna; painless, indolent swelling of both testicles; great muscular weakness, and entire inability to support the weight of the body on the lower extremities. These symptoms had commenced three months before. The child had been born perfectly well; and when about three weeks old, broke out with papular eruption, with successive pustules, and with ulcers at the angles of the eyes, mouth, and nose, preceded by snuffling and inability to nurse.

These symptoms were treated by another physician, with grey powders; and when three months old the child was apparently well. He continued well until the tenth month, when the present symptoms appeared, as already stated. The parents of the child have been married fifteen years. The father is a drinking man; confesses to have had primary symptoms before marriage, and has suffered much, and frequently from rheumatism and eruptions of the skin since, and has the latter at present. The mother is not aware that she ever had primary symptoms, and has had no recognized secondaries, except nocturnal rheumatic pains, from which she still suffers.

They have had four children before the present one, and all of them had well-marked eruptions and muscular weakness. They all died, at ages varying from three to fifteen months. This patient has been and is taking iodide of potassium, and the symptoms are all improving.—*N. Y. Med. Times.*

